# Editorial: Alternative approaches to antifungal drugs against drug-resistant fungi

**DOI:** 10.3389/fcimb.2023.1184922

**Published:** 2023-03-23

**Authors:** Renátó Kovács, Shahram Mahmoudi

**Affiliations:** ^1^Department of Medical Microbiology, Faculty of Medicine, University of Debrecen, Debrecen, Hungary; ^2^Department of Parasitology and Mycology, School of Medicine, Iran University of Medical Sciences, Tehran, Iran

**Keywords:** fungi, alternative therapeutic approach, *Candida*, *Aspergillus*, resistance

In the last two decades, the uncontrolled widespread usage of different antifungal agents in veterinary and human medicine as well as in agriculture has significantly changed the epidemiological landscape of invasive fungal infections, where fungal species showing multi – or panresistant isolates are steadily increasing. This worrisome trend can be observed in the worldwide emergence of difficult to treat azole-resistant *Aspergillus fumigatus*, *Candida auris* and multidrug-resistant *C. glabrata* (Arastehfar et al., 2020). Therefore, the World Health Organization published the list of fungal priority pathogens in 2022, which is the first global effort to systematically classify various fungal pathogens considering their current public health importance. In this report, *C. albicans*, *C. auris*, *A. fumigatus* and *Cryptococcus neoformans* were assigned to the critical priority group (WHO, 2022).

The number of novel antifungal drugs have been scarce over the last years, and only three agents can be found at least in phase 3 clinical trial such as rezafungin, oteseconazole and ibrexafungerp. The latter compound has been already approved in 2021 by the U.S. Food and Drug Administration for the treatment of vulvovaginal candidiasis (McCarty and Pappas). Therefore, it is necessary to discover and introduce new antifungal agents and/or alternative therapeutic approaches in clinical practice (Kovács and Majoros, 2020; Izadi et al., 2022; Lamoth, 2023).

This Special Issue addresses current innovative investigations regarding alternative antifungal therapies against potentially resistant fungal species. A total of four original articles were published in this Special Issue. Schwarz et al. tested the *in vitro* interaction of isavuconazole and colistin against various clinically relevant *Candida* species. Based on calculated fractional inhibitory concentration indices, the combination was synergistic in 50%, 80%, 90%, and 90% of the *C. kefyr*, *C. krusei*, *C. glabrata*, and *C. tropicalis* isolates tested, respectively. Isavuconazole with colistin against *C. albicans* and *C. parapsilosis* exhibited only indifferent interaction for 100% and 90% of the isolates, respectively. Moreover, antagonistic interaction was never observed. Plants are rich source of several aromatic bioactive compounds, which can facilitate the development of novel innovative antifungal strategies for the effective treatment of fungal pathogens. Regarding these compounds Lee et al. showed that curcumin triggered a decrease in Hsp90 by affecting it at the post-transcriptional level, which lead to the down-regulated level of *HOG1* and *CDR1* genes. This resulted in a decrease of the stress response and efflux pump activity as well as impair cell growth in *C. albicans*. In the past years, *Caesalpinia bonduc* has received increasing attention thanks to its wide spectrum antimicrobial effects. Sasidharan et al. examined ethanolic extracts of *C. bonduc* seeds (EECS) against four *Candida* species *in vitro* and *in vivo*. The EECS treatment exerted oxidative stress in *Candida* cells, which resulted the increase of cytoplasma membrane permeability of *C. albicans* and caused mitochondrial dysfunction. In *Galleria mellonella* model, the EECS treatment considerably increase the recovery rate of *G. mellonella* larvae following the treatment. The resistant phenotype is frequently associated with the biofilm forming ability. These sessile communities possess with higher resistance to environmental factors, immune response and antimicrobial agents (Ciofu et al., 2022). Regarding filamentous fungi, Sen et al. elucidated the antibiofilm properties of 4-allyl-2-methoxyphenol (eugenol) against ten soil-derived azole-resistant *A. fumigatus* isolates. Eugenol exhibited antibiofilm activity against these resistant isolates, ranging from 312 to 500 µg/mL. Surprisingly, the eugenol treatment was associated with absence of extracellular matrix of *A. fumigatus* biofilm. Furthermore, eugenol significantly decrease the transcription level of some efflux pumps genes including *MDR1* and *MDR4.*

In summary, this Special Issue is a great resource that presents novel research concerning alternative treatment strategies against clinically relevant fungal species. Hopefully, these published results will be able to stimulate further state-of-the-art studies in the future.

## Author contributions

RK and SM were guest associate editors of the Research Topic. They wrote and edited the paper text. All authors contributed to the article and approved the submitted version.
